# Circadian rhythm in turbot (*Scophthalmus maximus*): daily variation of blood metabolites in recirculating aquaculture systems

**DOI:** 10.1007/s11306-023-02077-9

**Published:** 2024-02-12

**Authors:** J. Petereit, G. Lannig, B. Baßmann, C. Bock, B. H. Buck

**Affiliations:** 1 Alfred Wegener Institute Helmholtz Centre for Polar and Marine Research (AWI), Am Handelshafen 12, 27570 Bremerhaven, Germany; 2 Faculty of Agricultural and Environmental Sciences, University of Rostock, Aquaculture and Sea-Ranching, Justus-Von-Liebig-Weg 6, 18059 Rostock, Germany; 3 University of Applied Sciences Bremerhaven, An Der Karlstadt 8, 27568 Bremerhaven, Germany

**Keywords:** Metabolism, NMR spectroscopy, Metabolomics, Daily rhythm, Stress physiology, Circadian timing

## Abstract

**Introduction:**

Animal welfare in aquaculture is becoming increasingly important, and detailed knowledge of the species concerned is essential for further optimization on farms. Every organism is controlled by an internal clock, the circadian rhythm, which is crucial for metabolic processes and is partially influenced by abiotic factors, making it important for aquaculture practices.

**Objective:**

In order to determine the circadian rhythm of adult turbot (*Scophthalmus maximus*), blood samples were collected over a 24-h period and plasma metabolite profiles were analyzed by ^1^H-NMR spectroscopy.

**Methods:**

The fish were habituated to feeding times at 9 am and 3 pm and with the NMR spectroscopy 46 metabolites could be identified, eight of which appeared to shift throughout the day.

**Results:**

We noted exceptionally high values around 3 pm for the amino acids isoleucine, leucine, valine, phenylalanine, lysine, and the stress indicator lactate. These metabolic peaks were interpreted as either habituation to the usual feeding time or as natural peak levels in turbot in a 24-h circle because other indicators for stress (glucose, cortisol and lysozymes) showed a stable baseline, indicating that the animals had no or very little stress during the experimental period.

**Conclusion:**

This study provides initial insights into the diurnal variation of metabolites in adult turbot; however, further studies are needed to confirm present findings of possible fluctuations in amino acids and sugars. Implementing optimized feeding times (with high levels of sugars and low levels of stress metabolites) could lead to less stress, fewer disease outbreaks and overall improved fish welfare in aquaculture facilities.

## Introduction

Fish is considered an important source of healthy food, and accurate information on best farming practices is required to optimize cultivation and ensure sustainable production (Ahmad et al., 2021; Bennett et al., 2021; FAO, 2022; Farmery et al., 2022). Animal welfare concerns often negatively influence consumer perceptions, especially in intensive fish farms (Ankamah-Yeboah et al., 2016; Funk et al., 2021; Maesano et al., 2020). Enhancing fish welfare is therefore essential to improve eco-intensification and increase the appeal of aquaculture products to consumers (Brijs et al., 2018).

The concept of animal welfare is a multilevel approach due to species dependency, high individual variability, and interrelated complex metabolic processes (Ashley, 2007; Carbonara et al., 2020; Martos-Sitcha et al., 2020; Toni et al., 2019; Zheng et al., 2021). There is not even one single measure for proof of welfare, but several and the most recognized indicators are immune response, stress level, and feeding behavior (Ashley, 2007; Toni et al., 2019; Tort, 2011). Fish have a circadian rhythm (CR) that controls these indicators and changes periodically depending on external factors such as light, temperature, or feeding times (Choi et al., 2020; Fortes-Silva et al., 2019; Frøland Steindal & Whitmore, 2019; Nikkhah, 2015; Sanchez-Vazquez et al., 2019). CR affects numerous cellular processes, including stress and immune responses as well as appetite in fish (Ceinos et al., 2019; Nikkhah, 2015; Pilorz et al., 2018; Prokkola & Nikinmaa, 2018; Zheng et al., 2021). It is well known in mammals that alteration of the feeding cycle can lead to a disruption in the circadian rhythm of the animal, in fish, however, the connections between the CR and feeding are largely unknown (Refinetti, 2015).

The consideration for the CR is therefore especially important for intensive aquaculture farms, which manipulate abiotic factors to increase efficiency, as well as work on fixed feeding schedules instead of adapting to the animals' metabolism in a species-specific manner (Callier et al., 2018; Choi et al., 2020; Pratiwy et al., 2021). Therefore, an optimized feeding regime regarding the CR of the respective species improves feed conversion without extra costs for the farmer, resulting in higher profitability. Moreover, avoiding feed waste through proper feeding minimizes the loss of expensive feed and the accumulation of nitrogenous waste in the water, which could be toxic to fish (Assan et al., 2021). Therefore, it is critical to evaluate the processes occurring over 24-h in order to adjust the feeding time (during stressed and non-stressed times of the day) to match the metabolism of the fish.

In intensive aquaculture, fish are exposed to many different stressors, such as inadequate stocking densities, handling, transportation, or restricted and unfamiliar environments (Assan et al., 2021; Martos-Sitcha et al., 2020). Recent studies have shown that handling fish at different times of the day is likely to result in better stress-coping mechanisms due to the daily fluctuations of stress metabolites in the organism (Figueiredo et al., 2020; Lopes et al., 2022; Montero et al., 2019). Stress is generally defined as a disruption of physiological or biological mechanisms caused by internal and external factors commonly referred to as stressors (Barton, 2002; Hernández-Pérez et al., 2019; Ramsay et al., 2009; Wendelaar Bonga, 1997). Many indicators of stress exist for fish in aquaculture, but plasma cortisol is one of the best-established and most frequently used in circadian rhythm studies (Cowan et al., 2017; Prokkola & Nikinmaa, 2018; Tort, 2011; Valenzuela et al., 2022). In addition to cortisol as a stress marker, glucose is of critical importance in aquaculture systems as it affects nutrient availability and oxygen consumption of animals (Prokkola & Nikinmaa, 2018). Moreover, glucose, along with other sugars, is the main energy supplier of organisms as it is converted to adenosine triphosphate (ATP) during cellular respiration (Cowan et al., 2017). This is important because the availability of energy in the organism helps the animal to cope better with stressful situations (Valenzuela et al., 2022). Stress furthermore leads to enhanced exercise in the animal and therefore generates more lactate by anaerobic glycolysis in the muscles (Fuller et al., 2020; Raposo de Magalhães et al., 2020). In stressful situations cortisol, lactate, and glucose are elevated in the blood of fish and are used as most common indicators for increased stress in fish (Herrera et al., 2019; Odhiambo et al., 2020).

Previous studies have shown a close relationship between immunological and stress responses in fish, which in turn can affect the interwoven pathways of the CR (Montero et al., 2019; Schleiermann et al., 2013; Valenzuela et al., 2022). Chronic stress may suppress immune responses and consequently increases the risk of disease outbreaks, especially on intensive fish farms (Hernández-Pérez et al., 2019; Sakai et al., 2021; Skouras et al., 2003; Song et al., 2021). A commonly used immune parameter is lysozyme since it is directly coupled to liver functions and decreased levels indicate high stress (Valenzuela et al., 2022). This has particular implications for fish health, and a better understanding could reduce the risk of disease outbreaks in aquaculture operations.

In general, a disruption of the daily rhythmicity in fish can lead to a temporal loss of homeostasis and consequently increases stress level, which in turn negatively affects energy availability, food intake, immune system and utilization (Cowan et al., 2017; Gómez-Boronat et al., 2018). To better understand these underlying processes in an organism, the use of metabolomics can help and allows the qualification of low molecular weight (< 1000 DA) metabolites (Cappello et al., 2019; Lin et al., 2006). Metabolomics based on NMR spectroscopy in the study of body fluids such as plasma and serum blood represent an effective approach to elucidate the interactions between aquatic organisms and their environment (Viant, 2007), discover biomarker profiles (Macias et al., 2019), and gain deep insights into mechanisms associated with circadian rhythms in fish (Figueiredo et al., 2020; Lopes et al., 2022; Montero et al., 2019).

Turbot (*Scophthalmus maximus L., 1758*) is a valuable marine fish species for aquaculture and has received remarkable attention in recent decades (Fraga-Corral et al., 2022; Hoerterer et al., 2022; Pyanov, 2021). Turbot is usually cultured in recirculating aquaculture systems (RAS) and land-based flow-through facilities, and the growth and health of this species are well studied, but information about their CR is scarce. This study focuses on the identification of metabolites in the blood of turbot kept in a RAS. No previous study has investigated the CR of blood metabolites in turbot. Such information is required in order to better understand the metabolism and make adjustments to aquaculture practices. Modifying feeding and handling times in intensive aquaculture to match species-specific CR could contribute to higher growth and better feed conversion. Consequently, these changes could lead to optimized farm profitability and improved fish welfare without imposing additional costs on the farm.

## Material and methods

### Experimental setup

A total of 60 turbot were reared at the ZAF (Centre for aquaculture research) of the Alfred Wegener Institute Helmholtz Centre for Polar and Marine Research (AWI) (Bremerhaven, Germany). The fish were acclimated in a RAS for two weeks before the experiment was started. The fish were randomly and evenly distributed among twelve tanks (5 fish/tank).

The experimental RAS consisted of 36 individual rearing tanks, each given a bottom size of 1 m^2^ and a volume of 700 l. For the experiment, fifteen of the 36 tanks were actively used, twelve for the experiment itself and three other tanks as a reserve and for replacement fish. The water was treated with standard RAS purification devices such as a drum filter, a biofilter, a protein skimmer (with ozone), and a trickling filter. Water parameters such as temperature, pH, salinity, oxygen were constantly monitored using a SC 1000 Multiparameter Universal Controller (Hach Lange GmbH, Düsseldorf, Germany). Nitrite, nitrate, and ammonium were measured twice a week using the QuAAtro39 AutoAnalyzer (SEAL Analytical, Germany). There were no deviations in water parameters during the entire experiment (Table 1).

**Table 1:** Mean values ± standard deviation of the water parameters (temperature, pH, salinity, and oxygen: n = 83; Ammonium, nitrite, and nitrate: n = 42)

Temperature (°C)	pH		Salinity	Oxygen (%)	Ammonium (mg/L)	Nitrite (mg/L)	Nitrate (mg/L)
20.0 ± 0.9	7.62 ± 0.04		36.3 ± 0.1	95.2 ± 6.7	0.096 ± 0.055	0.198 ± 0.11	127.9 ± 38

Twice a day at 9 am and 3 pm over a period of 6 months, the fish were fed with a commercial diet ad libitum in order to acclimate to the feeding times (see Hoerterer et al., 2022 for detailed information about diets and growth performances). Twenty-four hours before the sampling, the fish were starved (Jia et al., 2018). During the 6 month there was a 12 h light- dark cycle which the light turned on from 7am to 7 pm.

### Measurement and sampling

During the sampling, the tanks were randomly selected. From 7 am. on, the five fish of the first tank were netted and sampled, followed by the next tanks in a two-hour interval. This procedure was repeated until all the tanks were used, leading to a 24-h cycle (after Hernández-Pérez et al., 2019 and Lopes et al., 2022). The fish were netted, stunned by a blunt impact on the head, then killed by a neck cut. The sampling procedure was as follows: The fish were weighed, measured in length, and placed directly on ice. The blood was collected with a heparinized syringe from the caudal vessels (approximately 1 mL). The blood was stored in Eppendorf tubes and directly centrifuged at 2000 g at 7 °C for 10 min. The supernatant (plasma) was separated from the cells and stored at − 20 °C for further analysis. In order to ensure that metabolites were not further expressed, the entire process from netting to blood collection was kept within 5 min for all five fish together.

### Analysis of lysozyme activity

The lysozyme activity in the plasma was photometrically measured according to the protocol of Milla et al. (2010). The phosphate buffer consisted of 0.05 mol/L NaH2PO4 + 0.05 mol/L Na2HPO4 and was modified with 85% H3PO4 to a pH of 6.2 at room temperature. 30 mg of *Micrococcus luteus* (0.6 mg/mL, SIGMA M3770) were mixed with 50 mL buffer on a daily basis, while 20 mg lysozyme from egg whites (Lot SLCC4285, 40,382 Units/mg, Sigma L6876) were mixed with 20 mL of buffer weekly. The lysozyme-buffer solution was diluted to get 1000 U/mL. Therefore, 248 µL lysozyme solution was buffered in 9.752 mL buffer. A standard curve was created that ranged from 100 to 900 U/mL, as well as 2 blank samples (one with the bacterium and one with the buffer). For the samples, 10 µL and 5 µL of plasma with 10 µL and 15 µL of buffer were pipetted into the wells to obtain a volume of 20 µL per well. Immediately before starting the measurement, *M. luteus* was added with 130 µL to the standard curve and all sample preparations. For the measurement, 96 well plates (Brandplate 781,660) with clear flat bottom were used and measured in a Berthold Tristar LB941.

The measurement was performed at 450 nm every minute for ten minutes. The plate was shaken for five seconds before the first measurement. The standard curve, samples, and blanks were measured in triplicate for each measurement. Between measurements, the solutions were stored as long as possible in the refrigerator at 4 °C and the samples in the freezer at − 20 °C to stop the expression of further metabolic products as much as possible.

### Analysis of glucose, lactate and cortisol

Glucose and lactate were tested using the plasma samples on test strips (Accu-Check Aviva and Accutrend Plus, Roche, Mannheim, Germany). An enzyme-linked immunosorbent assay (ELISA, Cusabio, fish cortisol, sensitivity: 0.0023 ng/mL) was conducted according to the manufacturer's instructions to determine plasma cortisol. The absorbance was analyzed with a micro-plate reader at 450 nm (iMark, Bio-Rad, Feldkirchen, Germany).

### NMR analysis

Plasma samples were prepared by methanol extraction prior to untargeted metabolic profiling via NMR spectroscopy, according to Nagana Gowda and Raftery (2017).

Accordingly, 600 µL of plasma were mixed with 1200 µL of HPLC methanol and shaken for 30 s. Samples were first stored for 20 min at − 20 °C. Afterwards, the samples were centrifuged at 13,000 g at 3 °C for 30 min. The supernatant was transferred to a new vial and dried overnight in a vacuum concentrator (SpeedVac, RVC 2–33 IR, Christ GmbH, Germany). The dried samples were mixed with 600 µL of D_2_O (Sigma, Nr.775002) containing TSP standard (TSP; 0.05 wt%; Sigma Aldrich, St. Louis, USA) as an internal reference and centrifuged at 13,000 g at 7 °C for five minutes. 500 µl of the supernatant was transferred to a 5 mm NMR tube. The spectra were measured in an ultra-shielded vertical 9.4 T NMR spectrometer (Avance III HD 400 WB, Bruker-BioSpin GmbH, Germany) using a 5 mm BBx Probe at a proton frequency of 400 MHz. All samples were analyzed by one-dimensional ^1^H–NMR spectroscopy using a Carr-Purcell-Meiboom-Gill protocol with water presaturation at 21 °C (cpmg1pr) within TOPSPIN 3.2 software (TopSpin 3.5pl, Bruker-BioSpin GmbH, Germany).

Spectra were processed and analyzed using Chenomix NMR Suite 8.0 software (Chenomix nmr suite 8.4 Professional). Before analysis, all the spectra were manually corrected for phase, shim, and baseline and calibrated to the TSP standard. The metabolite peaks of the processed ranges were analyzed and assigned to their chemical compounds using the Chenomix database and literature as references (Beckonert et al., 2007; Götze et al., 2020; Rebelein et al., 2018). The concentrations of the assigned metabolites were determined by the Chenomix software based on the concentration of the internal standard (TSP set to 3.2 mM).

### Statistical analysis

Plasma cortisol, glucose, and lactate were analyzed using Sigma Plot (12.5, Systat Software, Germany). All data was tested successfully for normality and homogeneity of variances. Afterwards, a One-way analysis of variance (ANOVA) was performed to detect significant differences between time periods (p < 0.05). The results were expressed as means with standard deviations.

Statistics on metabolite profiles of NMR spectra were performed using the online platform MetaboAnalyst 5.0 (https://www.metaboanalyst.ca/, after Xia & Wishart, 2016). Metabolites were normalized by cubic root transformation and autoscaling to stabilize the variation between metabolites. Then, one-way ANOVA was performed with Fisher's LSD post hoc test at an adjusted p value of 0.05. In addition, significance analysis of metabolites (SAM) was performed with equal group variances and a false discovery rate (FDR) of 0.3. Significant differences between groups of altered metabolites were plotted using Sigma Plot (12.0, Systat Software, Inc.). Unsupervised principal component analysis (PCA) together with heatmaps were performed for the identification of potential outliers (not shown).

## Results

The fish had a weight of 551.94 g ± 102 g and a length of 30.79 cm ± 2.06 cm. No deaths of the fish or any other problem occurred during the sampling day (24 h).

### Analysis of lysozyme, cortisol, glucose and lactate

**Fig. 1:**
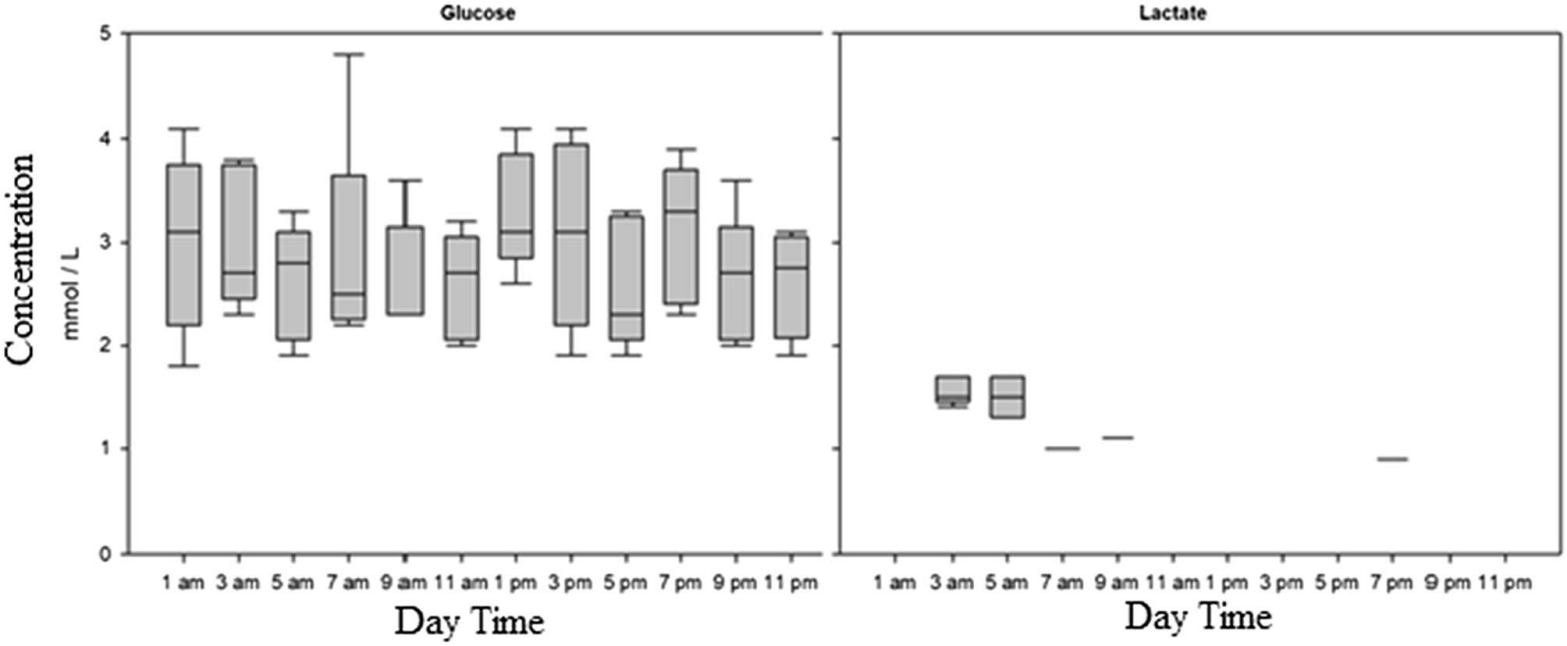
Glucose and lactate levels in the plasma of *Scophthalmus maximus* related to the specific time of the day. Data are mean concentrations in mmol / L ± SD, n=5

**Fig. 2:**
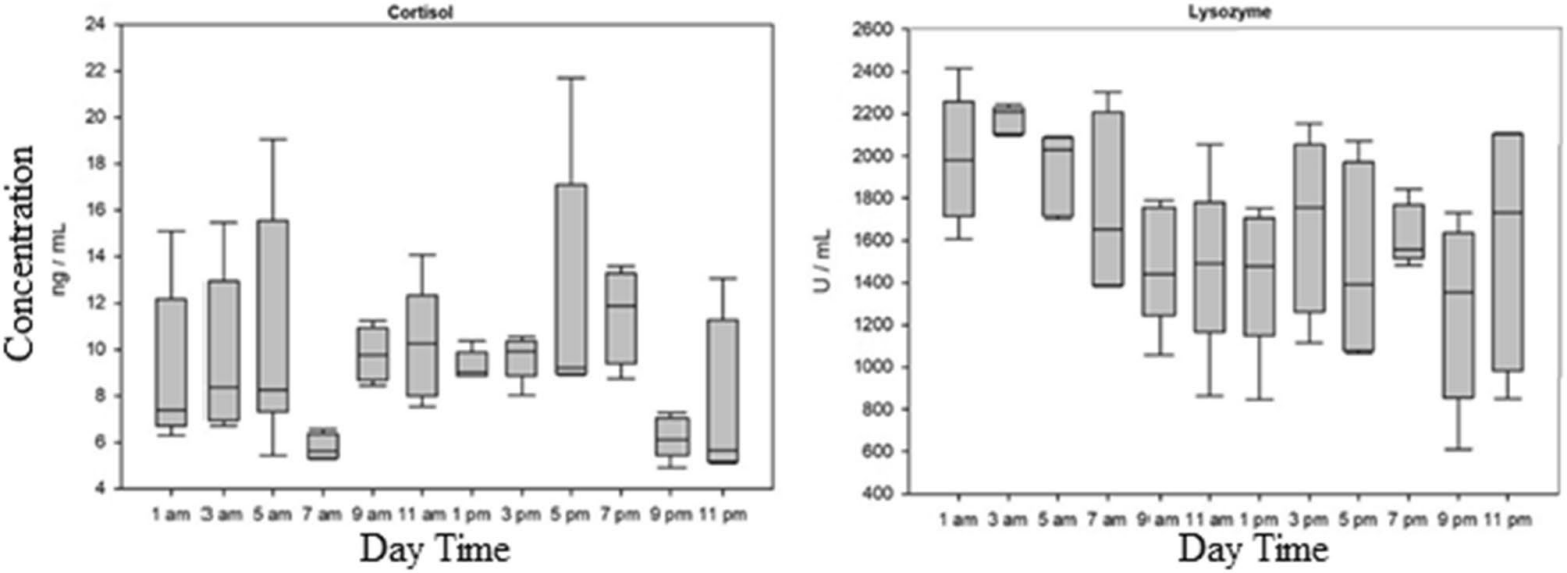
Cortisol und lysozyme levels in the plasma of *Scophthalmus maximus* related to the specific time of the day. Data are mean concentrations in ng/mL for cortisol and U/ mL for lysozymes ± SD, n=5

The results show no significant differences in glucose, lysozyme, and cortisol between the timepoints (Fig. 1 and 2, Annex: Table 2). The lactate levels changed over the 24 h sampling time and were elevated at 3 am and 5 am (Fig. 1). Most lactate levels were below the lower threshold of 0.7 mmol/L and could not be detected exactly with the test stripes.

**Table 2:** Plasma metabolites n = 5 given as mean values with standard deviation

Time/Metabolite (mM)	1:00 AM	3:00 AM	5:00 AM	7:00 AM	9:00 AM	11:00 AM	1:00 PM	3:00 PM	5:00 PM	7:00 PM	9:00 PM	11:00 PM
Glucose (mmol/L)	3.00	3.02	2.62	2.86	2.64	2.58	3.30	3.08	2.58	3.10	2.62	2.63
± SD	0.77	0.61	0.50	0.98	0.50	0.46	0.51	0.81	0.56	0.61	0.57	0.46
Lactate (mmol/L)	bT	1.56	1.50	1.00	bT	bT	bT	bT	bT	0.90	bT	bT
± SD		0.12	0.20	0.00						0.00		
Lysozyme (U/mL)	1983.09	2174.05	1926.73	1748.52	1384.39	1477.27	1438.55	1677.73	1498.27	1625.36	1267.27	1606.08
± SD	271.46	59.63	174.34	386.78	259.88	378.09	316.82	373.86	407.74	128.75	388.96	526.94
Cortisol (ng/mL)	9.23	9.83	10.50	5.66	9.86	10.17	9.24	9.63	12.48	11.54	6.14	7.74
± SD	3.45	4.00	5.11	1.09	2.20	3.53	0.97	1.41	5.41	2.15	1.25	3.68

Grey highlighted numbers represent the timepoint with the highest concentration for that metabolite

*Legend* bT—> below Threshold

### Metabolic profiling with NMR spectroscopy

Forty Six metabolites could be identified in the ^1^H-NMR spectra from the plasma of turbot (Table 3 in the Annex).

**Table 3:** Plasma Metabolites (n = 5) in mM given as mean values ± SD at the different timepoints

Time/Metabolite (mM)	1:00 AM	3:00 AM	5:00 AM	7:00 AM	9:00 AM	11:00 AM	1:00 PM	3:00 PM	5:00 PM	7:00 PM	9:00 PM	11:00 PM
Acetate	0.408	0.293	0.387	0.438	0.459	0.405	0.400	1.102	0.616	0.491	0.469	0.443
± SD	0.055	0.043	0.015	0.056	0.073	0.088	0.046	0.744	0.273	0.370	0.091	0.091
Alanine	0.260	0.154	0.229	0.247	0.261	0.271	0.253	0.479	0.369	0.295	0.473	0.299
± SD	0.091	0.032	0.065	0.056	0.039	0.059	0.047	0.284	0.202	0.188	0.278	0.089
Asparagine	0.198	0.115	0.103	0.071	0.194	0.080	0.066	0.130	0.171	0.054	0.060	0.154
± SD	0.184	0.149	0.115	0.033	0.231	0.032	0.046	0.113	0.121	0.031	0.048	0.193
Aspartate	0.189	0.038	0.190	0.104	0.114	0.065	0.070	0.526	0.207	0.234	0.098	0.148
± SD	0.165	0.034	0.141	0.108	0.085	0.055	0.064	0.706	0.117	0.403	0.074	0.200
Betaine	0.040	0.015	0.022	0.034	0.023	0.027	0.047	0.064	0.036	0.048	0.030	0.044
± SD	0.024	0.009	0.013	0.014	0.007	0.015	0.014	0.035	0.021	0.039	0.008	0.026
Biotin	0.050	0.037	0.061	0.072	0.089	0.060	0.083	0.104	0.092	0.074	0.058	0.060
± SD	0.001	0.020	0.029	0.002	0.029	0.021	0.050	0.080	0.068	0.006	0.013	0.022
Carnitine	0.002	0.026	0.017	0.004	0.030	0.029	0.026	0.089	0.035	0.004	0.023	0.026
± SD	0.013	0.013	0.010	0.004	0.006	0.012	0.006	0.070	0.017	0.030	0.016	0.012
Choline	0.031	0.023	0.024	0.030	0.004	0.026	0.022	0.034	0.003	0.029	0.020	0.035
± SD	0.011	0.005	0.009	0.011	0.035	0.013	0.008	0.021	0.011	0.013	0.006	0.027
Citrate	0.059	0.049	0.041	0.066	0.068	0.043	0.043	0.070	0.058	0.044	0.044	0.056
± SD	0.035	0.044	0.008	0.038	0.040	0.020	0.009	0.047	0.026	0.035	0.024	0.026
Creatine	0.076	0.055	0.070	0.080	0.086	0.062	0.057	0.281	0.145	0.069	0.069	0.089
± SD	0.018	0.034	72.100	0.028	0.029	0.026	0.013	0.282	0.071	0.020	0.025	0.041
Creatine phosphate	0.141	0.084	0.114	0.136	0.147	0.102	0.121	0.461	0.236	0.125	0.105	0.169
± SD	0.021	0.050	0.030	0.048	0.039	0.026	0.060	0.522	0.139	0.048	0.036	0.125
Creatinine	0.069	0.052	0.052	0.098	0.082	0.054	0.068	0.130	0.115	0.046	0.051	0.077
± SD	0.010	0.027	0.011	0.030	0.017	0.026	0.034	0.136	0.075	0.024	0.017	0.051
Fumarate	0.002	0.001	0.003	0.002	0.002	0.002	0.003	0.004	0.002	0.003	0.002	0.002
± SD	0.001	0.000	0.002	0.002	0.002	0.000	0.002	0.002	0.001	0.001	0.001	0.001
Glucose	1.978	1.930	1.599	1.965	1.994	1.951	1.968	3.641	2.920	2.038	1.872	2.050
± SD	0.534	0.572	0.339	0.501	0.355	0.701	0.940	2.472	1.220	0.877	0.271	0.315
Glutamate	0.246	0.166	0.222	0.254	0.272	0.234	0.247	0.608	0.310	0.361	0.240	0.247
± SD	0.032	0.092	0.058	0.061	0.074	0.051	0.078	0.524	0.107	0.244	0.059	0.127
Glutamine	0.120	0.083	0.133	0.136	0.151	0.087	0.122	0.209	0.145	0.124	0.152	0.111
± SD	0.037	0.042	0.024	0.019	0.031	0.015	0.050	0.164	0.052	0.102	0.037	0.070
Glycine	0.223	0.204	0.192	0.249	0.273	0.223	0.216	0.595	0.280	0.264	0.255	0.303
± SD	0.064	0.051	0.058	0.044	0.122	0.077	0.052	0.466	0.080	0.200	0.120	0.158
Histamine	0.010	0.008	0.013	0.015	0.012	0.011	0.012	0.018	0.009	0.008	0.007	0.008
± SD	0.004	0.001	0.007	0.014	0.004	0.003	0.005	0.006	0.001	0.003	0.001	0.001
Homocysteine	0.084	0.031	0.039	0.086	0.060	0.034	0.046	0.096	0.050	0.058	0.041	0.071
± SD	0.095	0.019	0.014	0.115	0.069	0.026	0.018	0.117	0.033	0.062	0.021	0.040
Isoleucine	0.134	0.092	0.129	0.294	0.263	0.196	0.249	0.343	0.292	0.278	0.188	0.204
± SD	0.048	0.033	0.037	0.090	0.064	0.054	0.044	0.251	0.114	0.152	0.028	0.067
Lactate	0.241	0.159	0.225	0.265	0.281	0.258	0.241	1.093	0.300	0.372	0.400	0.355
± SD	0.082	0.045	0.061	0.032	0.034	0.067	0.073	0.849	0.108	0.310	0.329	0.181
L-Arginine	0.482	0.377	0.451	0.527	0.623	0.491	0.524	0.706	0.595	0.455	0.310	0.521
± SD	0.131	0.153	0.196	0.118	0.128	0.141	0.103	0.370	0.122	0.098	0.130	0.184
Leucine	0.537	0.359	0.430	0.783	0.698	0.542	0.685	1.050	0.918	0.860	0.583	0.691
± SD	0.085	0.114	0.176	0.233	0.112	0.133	0.117	0.532	0.364	0.389	0.090	0.288
Lysine	0.149	0.078	0.145	0.158	0.192	0.147	0.168	0.516	0.183	0.083	0.155	0.139
± SD	0.039	0.017	0.034	0.040	0.033	0.039	0.012	0.426	0.013	0.041	0.017	0.056
Malate	0.111	0.087	0.098	0.108	0.142	0.098	0.103	0.166	0.149	0.094	0.083	0.110
± SD	0.032	0.050	0.025	0.010	0.044	0.038	0.015	0.091	0.064	0.055	0.031	0.028
Maltose	0.107	0.120	0.064	0.087	0.163	0.042	0.070	0.227	0.069	0.056	0.067	0.171
± SD	0.080	0.106	0.020	0.077	0.216	0.013	0.048	0.183	0.019	0.022	0.025	0.236
Mannitol	0.579	0.442	0.553	0.627	0.667	0.657	0.786	1.830	0.946	0.700	0.841	0.794
± SD	0.256	0.144	0.169	0.284	0.120	0.144	0.051	1.230	0.521	0.463	0.241	0.185
Methionine	0.061	0.043	0.082	0.092	0.106	0.076	0.109	0.218	0.135	0.097	0.085	0.114
± SD	0.024	0.029	0.027	0.033	0.040	0.032	0.037	0.177	0.094	0.092	0.021	0.027
Methylamine	0.052	0.034	0.049	0.046	0.055	0.032	0.035	0.094	0.058	0.045	0.037	0.051
± SD	0.029	0.029	0.031	0.021	0.033	0.007	0.012	0.092	0.023	0.055	0.011	0.033
O-Acetyl choline	0.038	0.032	0.027	0.042	0.031	0.029	0.037	0.098	0.039	0.045	0.025	0.036
± SD	0.009	0.011	0.006	0.008	0.003	0.005	0.027	0.077	0.009	0.024	0.009	0.012
Ornithine	0.074	0.070	0.069	0.074	0.070	0.060	0.060	0.061	0.069	0.063	0.063	0.068
± SD	0.005	0.025	0.010	0.008	0.009	0.021	0.026	0.020	0.009	0.016	0.013	0.012
Phenylalanine	0.065	0.045	0.041	0.148	0.106	0.100	0.151	0.214	0.114	0.101	0.062	0.084
± SD	0.024	0.025	0.023	0.055	0.048	0.040	0.035	0.183	0.067	0.051	0.020	0.016
Pipecolate	0.070	0.053	0.103	0.090	0.113	0.142	0.067	0.354	0.125	0.137	0.147	0.078
± SD	0.011	0.058	0.023	0.023	0.052	0.123	0.023	0.491	0.052	0.159	0.074	0.027
Pyro glutamate	0.086	0.084	0.102	0.142	0.130	0.100	0.145	0.112	0.144	0.142	0.076	0.100
± SD	0.042	0.077	0.052	0.017	0.016	0.023	0.093	0.036	0.024	0.111	0.031	0.040
Pyruvate	0.029	0.022	0.028	0.044	0.036	0.035	0.028	0.140	0.043	0.039	0.034	0.039
± SD	0.009	0.011	0.014	0.013	0.011	0.016	0.009	0.163	0.026	0.018	0.013	0.032
Serotonin	0.037	0.031	0.037	0.051	0.070	0.049	0.044	0.085	0.046	0.050	0.043	0.034
± SD	0.012	0.009	0.010	0.010	0.030	0.011	0.006	0.076	0.014	0.012	0.008	0.013
Succinate	0.017	0.013	0.015	0.028	0.020	0.013	0.016	0.070	0.020	0.016	0.018	0.018
± SD	0.005	0.007	0.007	0.008	0.007	0.005	0.006	0.073	0.015	0.007	0.008	0.012
Succinyl acetone	0.043	0.024	0.044	0.056	0.048	0.034	0.045	0.037	0.046	0.031	0.044	0.040
± SD	0.003	0.010	0.009	0.022	0.016	0.015	0.010	0.019	0.005	0.010	0.014	0.008
Sucrose	0.035	0.016	0.011	0.009	0.069	0.010	0.012	0.060	0.018	0.019	0.012	0.032
± SD	0.031	0.010	0.003	0.004	0.134	0.005	0.006	0.052	0.009	0.016	0.005	0.037
Taurine	0.083	0.129	0.091	0.091	0.072	0.077	0.104	0.079	0.060	0.126	0.073	0.101
± SD	0.020	0.049	0.013	0.046	0.028	0.032	0.052	0.029	0.038	0.070	0.016	0.034
Trimethyl amine N-oxide	0.025	0.012	0.017	0.025	0.025	0.018	0.032	0.037	0.021	0.042	0.017	0.016
± SD	0.014	0.006	0.006	0.006	0.010	0.012	0.017	0.030	0.008	0.056	0.005	0.009
Tryptophan	0.015	0.014	0.023	0.034	0.020	0.017	0.027	0.022	0.015	0.023	0.013	0.018
± SD	0.004	0.004	0.013	0.027	0.005	0.006	0.016	0.014	0.006	0.010	0.003	0.003
Tyramine	0.029	0.027	0.035	0.059	0.058	0.026	0.042	0.081	0.045	0.039	0.034	0.044
± SD	0.012	0.013	0.009	0.020	0.026	0.014	0.022	0.090	0.041	0.020	0.026	0.015
Tyrosine	0.037	0.045	0.048	0.048	0.045	0.070	0.073	0.088	0.066	0.059	0.036	0.057
± SD	0.021	0.024	0.037	0.010	0.016	0.025	0.036	0.067	0.029	0.037	0.009	0.007
Uracil	0.008	0.006	0.008	0.008	0.008	0.010	0.009	0.017	0.009	0.007	0.008	0.007
± SD	0.002	0.001	0.003	0.004	0.001	0.001	0.001	0.013	0.002	0.000	0.001	0.001
Valine	0.259	0.171	0.207	0.483	0.428	0.301	0.394	0.808	0.459	0.430	0.295	0.294
± SD	0.072	0.055	0.050	0.167	0.078	0.084	0.096	0.515	0.157	0.147	0.040	0.103

The main compounds were osmolytes and amino acids. Organic osmolytes were taurine, betaine, methylamine, and trimethyl-N-oxide (TMAO). Free amino acids identified were alanine, asparagine, aspartate, glutamine, glutamate, glycine, homocysteine, isoleucine, L-arginine, leucine, lysine, methionine, ornithine, phenylalanine, tryptophan, and valine, as well as amino acid derivatives such as pyroglutamate, pipecolate, succinyl acetone, tyrosine, and uracil. Biotin, a vitamin, was found in very low concentrations. Equally low concentrations were found for carnithine. creatine, creatinine and creatine phosphate, as well as in four compounds of the Krebs cycle, namely citrate, succinate, malate and fumarate. We also found glucose and pyruvate as indicators of glycolysis and energy storage. In addition, we identified one membrane-related intermediate, choline, and two sugars: maltose, sucrose, as well as the sugar alcohol mannitol. Metabolites involved in immune and stress responses found were histamine, lactate, O-acetylcholine, and serotonin. The singlet of acetate, involved in appetite in fish, was assigned despite overlap with arginine and lysine.

**Fig. 3:**
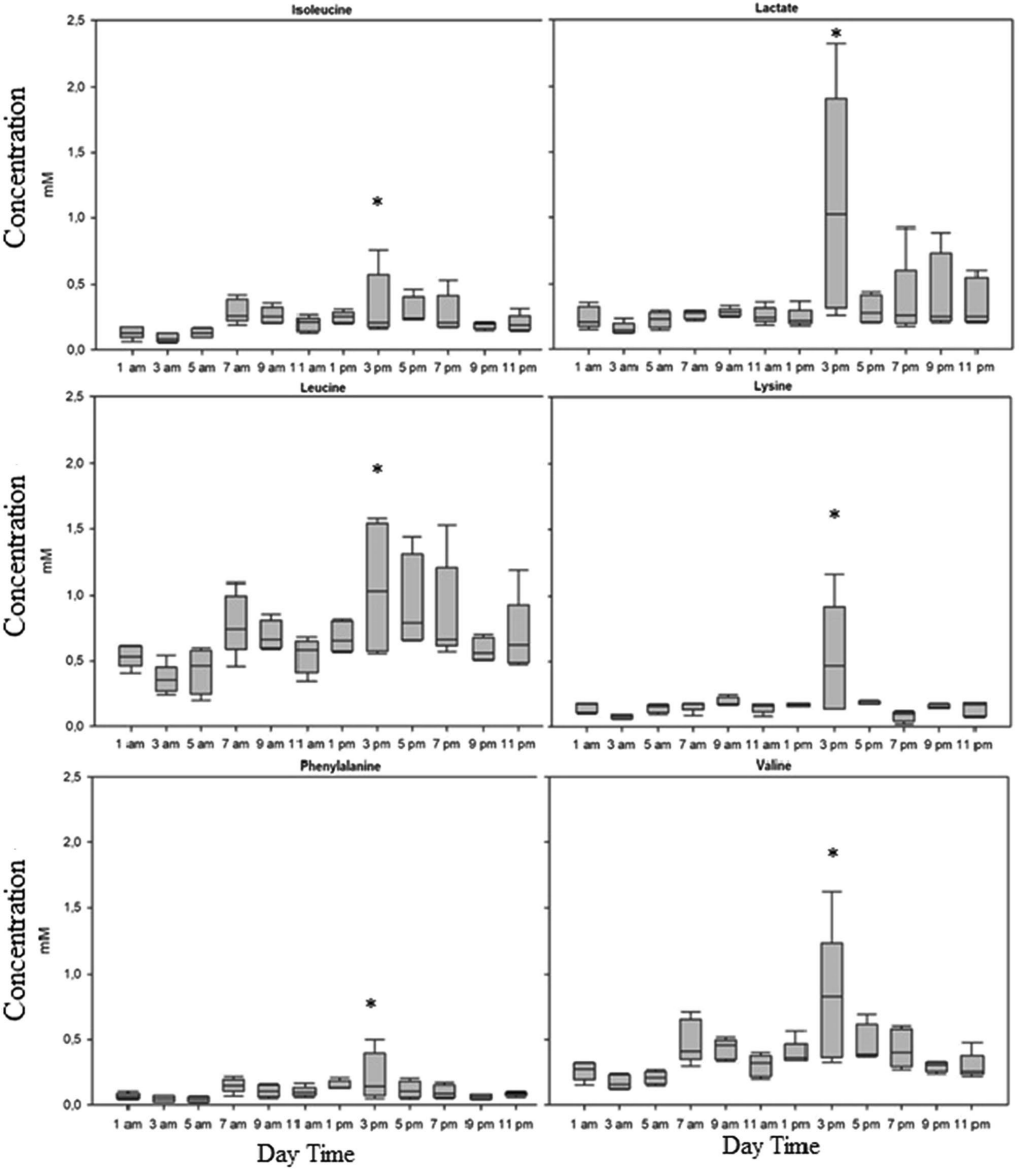
Isoleucine, lactate, leucine, lysine, phenylalanine and valine levels in plasma of *Scophthalmus maximus* related to the specific time of the day. Data are mean concentrations in mM ± SD, n=5, significantly elevated values are indicated with *

The number of metabolites did not change between samples, but significant sampling-time-dependent variations in metabolite concentrations could be observed. The One-Way ANOVA (p < 0.05) and SAM (Delta = 0.3) results show significantly elevated levels for isoleucine, leucine, valine, phenylalanine, lysine, and lactate at 3 pm within a 24-h period (Fig. 3).

## Discussion

In addition to commonly used stress parameters such as levels of cortisol, lactate and glucose, we applied untargeted metabolic profiling based on NMR spectroscopy to identify metabolic changes in turbot during a 24-h period and found some initial insights into the diurnal variation of metabolites in adult turbot. For better clarity and understanding of this complex issue, metabolites were divided into the three categories: indicators of stress responses, immune functions and amino acids.

### Stress response

Cortisol, glucose, and lactate are well-known and well-established primary and secondary stress indicators, and the concentrations determined here are within the range of previously published work (Deborde et al., 2021; Foss et al., 2019; Hernández- Pérez et al., 2019; Imsland et al., 2008; López-Olmeda et al., 2009; Salamanca et al., 2020; van Ham et al., 2003).

This study shows no significant differences in cortisol concentrations during the 24-h period examined, which is consistent with studies of gilthead sea bream (*Sparus aurata*), Senegalese sole (*Solea senegalensis*) (Costas et al., 2011), trout (*Oncorhynchus mykiss*) and turbot (*Scophthalmus maximus*) (Hernández-Pérez et al., 2019; Montoya et al., 2010; Nagel et al., 2012). However, in line with other studies (e.g. trout (Hernández-Pérez et al., 2019) or sea bream (López-Olmeda et al., 2009)) somewhat lower levels were found shortly after lights were turned on (7 am) and off (9 pm), suggesting that overall stress levels appear to be lowest at these times. Other studies have shown that cortisol levels follow a diurnal pattern and may indicate the time when fish are least stressed according to their rhythms (Figueiredo et al., 2020; Hernández-Pérez et al., 2019; Lopes et al., 2022; Montoya et al., 2010; Sánchez-Vázquez et al., 2019). As this is the first study to investigate cortisol levels in turbot over 24 h in addition with a rather low data record, further studies are needed to confirm our results.

In the present study, the glucose values show that turbot have a very stable baseline. This is not surprising, as other studies have indicated an identical pattern for glucose with similar values (1.2 mmol/ L—5.1 mmol/L), as in trout (*Oncorhynchus mykiss*), Senegalese sole (*Solea senegalensis*) or catfish (*Lophiosilurus alexandri*) (Costas et al., 2011; Figueiredo et al., 2020; Fortes-Silva et al., 2019; Hernández-Pérez et al., 2019; López-Olmeda et al., 2009). At 3 pm only, a slight increase in glucose, maltose, and sucrose was observed, which may indicate that fish have become accustomed to feeding at this time. Studies have shown that blood glucose levels of fish increase after their usual feeding time (Hernández-Pérez et al., 2019). According to Lopes et al. (2022) peak hormone and glucose activities are related to feeding cycles. Since our fish were subjected to a 24-h fasting period which may have prevented a clear pattern from forming further experiments testing the impact of starvation duration might be helpful. In addition, future studies with automatic feeders and feeding at other times of the day could show whether 3 pm is an important time for feeding turbot. If the fish are used to feeding at a certain time, the results could also be interpreted as an anticipatory response. If these results were reconstructed with fish that are not used to feeding at 3 pm, but at 1 pm and 5 pm, it could be concluded that 3 pm would be the best time to feed turbot due to lower stress responses.

Overall, stress responses in fish from aquaculture farms are poorly understood and require further attention (Figueiredo et al., 2020). Nonetheless, identifying stress variations may be an interesting tool to minimize the effects of stressors in an aquaculture farm and thus improve fish welfare. Studies in gilthead sea bream and catfish have shown that feeding times influence the level of cortisol in relation to diurnal rhythms, further increasing the importance of underlying processes in identifying optimal species-specific feeding times (Fortes-Silva et al., 2019; Montoya et al., 2010).

### Immunological response

Identifying the lowest immunological barriers is important to optimize feeding, transport, and handling in RAS and reduce the risk of disease outbreaks in aquaculture operations. Several studies suggest a strong link between the immune response and the circadian rhythm in fish, which is often influenced by light and feeding times (Costas et al., 2011; Montero et al., 2019; Scheiermann et al., 2013; Tort, 2011). However, in this study, no significant differences in the immunological parameters lysozyme, histamine, O- acetylcholine, or serotonin were detected within a 24-h period in turbot. Lysozyme levels were within the range of other studies (551–2194 U/mL) and were slightly higher between 1 and 5 am, indicating the best immune functionality (Grinde et al., 1988; Sun et al., 2016). This can be explained by the fact that the turbot was not stressed by light, feeding or handling at these times and were in a resting state.

In addition to assessing stress, lactate also serves as an important immune parameter and high lactate levels in fish could increase the risk of disease (Jääskeläinen et al., 2019). A suggestion for future aquaculture operations could therefore be to stop feeding at high lactate levels in turbot to prevent risk of disease outbreaks. In the lactate test strips, significantly higher levels were observed in turbot between 3 and 9 am. However, it is questionable how accurate these values are, even though they are within the range of lactate in turbot blood (0.6–1.1 mM) (Costas et al., 2011). The concentrations are often below the threshold of the strips and the values could not be confirmed with NMR spectroscopy. Because glucose was detected in both the strips and NMR, we could interpret that the lactate levels were too low for determination with the strips and, therefore could not be detected. The trend in NMR data of lactate cannot be reproduced with the lactate strips and must therefore be interpreted with caution. Further studies need to reproduce this trend, in which we found elevated lactate levels in the test strips between 3 and 9 am but elevated levels in NMR spectroscopy at 3 pm. Identifying the lowest immunological barriers is important to optimize feeding, transport, and handling in RAS and reduce the risk of disease outbreaks in aquaculture.

### Amino acids and energy metabolism

Amino acids (AAs) show a strong correlation with stress and immune parameters in fish and can help to better cope with stressful situations (Costas et al., 2011). All measured levels of AAs were within the range of fish and the most important ones will be discussed in more detail (*Solea senegalensis*, Costas et al., 2011). Branched-chain amino acids (BCAAs) are essential for regulating protein synthesis in skeletal muscle and are crucial to fish physiology (Ahmad et al., 2021). BCAAs include isoleucine (0.00–20.0 mM), valine (0.21–14.0 mM), and leucine (0.35–4.8 mM), all of which have values that are within the normal range for fish blood (Ahmad et al., 2021). All three BCAAs share the same intestinal transport and catabolic enzyme system, resulting in a high correlation. Both deficiency and excess of leucine and isoleucine in the diet can lead to reduced growth in many fish species and, therefore the optimization of BCAAs in fish diets can be critical for maximizing fish growth and health (Ahmad et al., 2021; Ahmed & Khan, 2004; Deng et al., 2014; Yamamoto et al., 2004). In this study, day time-dependent changes were found in BCAA concentrations, which seem to increase during the day (from 7 am to 7 pm, when it is light), and show the highest level at 3 pm. These results are supported by other studies that showed that light significantly affects the BCAA concentrations (Wu et al., 2021). Implementations for the increased concentrations at 3 pm could be to feed the animals during this period to optimize growth and digestion.

Additionally levels of phenylalanine (0.21–0.37 mM) and lysine (0.14–0.46 mM) were also significantly increased at 3 pm and were within the normal range for fish blood (Regost et al., 1999; Wei et al., 2020). Phenylalanine is a glycogenic essential amino acid (EAA) and is responsible for many metabolic functions such as feed conversion, antioxidant capacity, or protein storage capacity (Xiao et al., 2020). Most aquaculture feeds include alternative protein sources to fishmeal. Often plant-derived proteins are introduced into feed formulations but do not meet the essential amino acid requirements for fish (Petereit et al., 2022; Xiao et al., 2020). A recent study by Salamanca et al. (2020) provided information that adding tyrosine, the precursor of phenylalanine, and phenylalanine to the diet leads to less stressed fish under typical stress conditions in aquaculture. Our data show peaks of both metabolites at 3 pm. Using this information and comparing it to the natural occurrence of phenylalanine in other fish species may improve turbot welfare as well as feed utilization under standard aquaculture practices without adding expensive additives to alternative plast based diets (Xiao et al., 2020).

Lysine is another EAA and is usually the limiting factor in plant-based fish feed ingredients (Ebeneezar et al., 2019). A deficiency of lysine in the diet impairs the immunity of animals and increases their susceptibility to infectious diseases (Liao et al., 2015). In our study, as with many other essential AAs, a peak was detected at 3 pm, suggesting that this is a good time for feeding or handling. The natural occurrence of essential amino acids at 3 pm could promote feed conversion and thus fish growth and profitability. Stress conditions overall favor the mobilization of AA towards the fish brain and supplementations of AA could be used as energy substrates to better cope with stress (Costas et al., 2011; Salamanca et al., 2020).

## Conclusion

Changed circadian rhythms in aquaculture farms are a growing concern that impacts candidate’s health, food safety and security, and farm profitability. For the first time the daily rhythm of turbot was investigated in regard to plasma metabolites.

46 metabolites could be identified using NMR Spectroscopy, eight of which appeared to shift throughout the day. We noted an increase of metabolites around 3 pm, including several sugars, like glucose and sucrose as well as multiple amino acids like phenylalanine, leucine, isoleucine, valine and lysine. All are known as energy suppliers and using times of high metabolic rates for feeding could help to decrease the affection of stressful situations in fish. We furthermore saw no significant changes in the immune response over the day, neither in lysozymes nor cortisol. Previous studies did already show a correlation between immune function, stress and feeding ratio, which needs to be elaborated for turbot to optimize their farming practices. Further studies are needed to provide a deeper insight into the CR of turbot to help identify best feeding and handling times. A possible experimental design could include shorter sampling periods (e.g., every hour instead of every two hours), more experimental animals, exclusion of the 24-h starvation period, and multiple sample days for better comparison. This could potentially provide complementary data on whether the animals are accustomed to feeding at 3 pm and metabolite levels are elevated because of it or whether it is part of the natural rhythm of the fish.

The data collected may support rearing protocols and improve the overall health of turbot in aquaculture. This would not only have a positive impact on farm productivity but also lead to significant improvements in economic and animal welfare terms.

## Data Availability

Not applicable.
